# The dynamic risk factors of cardiovascular disease among people living with HIV: a real-world data study

**DOI:** 10.1186/s12889-024-18672-x

**Published:** 2024-04-25

**Authors:** Ziang Liu, Jiajia Zhang, Xueying Yang, Haoyuan Gao, Shujie Chen, Sharon Weissman, Bankole Olatosi, Xiaoming LI

**Affiliations:** 1 Department of Epidemiology and Biostatistics, Arnold School of Public Health, University of South Carolina, 915 Greene Street, Columbia, SC 29208 USA; 2 Arnold School of Public Health, South Carolina SmartState Center for Healthcare Quality, University of South Carolina, Columbia, SC 29208 USA; 3 Department of Health Promotion, Education and Behavior, Arnold School of Public Health, University of South Carolina, Columbia, SC 29208 USA; 4 Department of Internal Medicine, School of Medicine, University of South Carolina, Columbia, SC 29208 USA; 5 Department of Health Services Policy and Management, Arnold School of Public Health, University of South Carolina, Columbia, SC 29208 USA

**Keywords:** Cardiovascular Disease, HIV, Chronic disease, CD4 count, Viral load, South Carolina

## Abstract

**Background:**

This study aims to investigate the incidence and dynamic risk factors for cardiovascular diseases (CVD) among people living with HIV (PLWH).

**Methods:**

In this population-based statewide cohort study, we utilized integrated electronic health records data to identify adult (age ≥ 18) who were diagnosed with HIV between 2006 and 2019 and were CVD event-free at the HIV diagnosis in South Carolina. The associations of HIV-related factors and traditional risk factors with the CVD incidence were investigated during the overall study period, and by different follow-up periods (i.e., 0-5yrs, 6-10yrs 11-15yrs) using multivariable logistic regression models.

**Results:**

Among 9,082 eligible participants, the incidence of CVD was 18.64 cases per 1000 person-years. Overall, conventional risk factors, such as tobacco use, hypertension, obesity, chronic kidney disease (CKD), were persistently associated with the outcome across all three groups. While HIV-related factors, such as recent CD4 count (e.g., > 350 vs. <200 cells/mm^3^: adjusted odds ratio [aOR] range: 0.18–0.25), and percent of years in retention (e.g., 31–75% vs. 0–30%: aOR range: 0.24–0.57) were associated with lower odds of CVD incidence regardless of different follow up periods. The impact of the percent of days with viral suppression gradually diminished as the follow-up period increased.

**Conclusions:**

Maintaining an optimal viral suppression might prevent CVD incidence in the short term, whereas restoring immune recovery may be beneficial for reducing CVD risk regardless of the duration of HIV diagnosis. Our findings suggest the necessity of conducting more targeted interventions during different periods of HIV infection.

**Supplementary Information:**

The online version contains supplementary material available at 10.1186/s12889-024-18672-x.

## Background

People living with HIV (PLWH) had a prolonged life expectancy with the improvement and widespread use of antiretroviral therapy (ART) [1]. As a result of their aging process, chronic conditions, such as cardiovascular disease (CVD), diabetes, and hypertension, have become critical healthcare issues among PLWH [2–4]. Given the persistent increase of CVD incidence, CVD mortality is becoming a leading cause of death in PLWH. An increased focus is needed on the prevention and treatment of CVD for PLWH [5, 6]. According to the reports from several cohort studies in the US, it is estimated that the incidence rate of CVD among PLWH ranges from 2.57 to 7.10 cases per 1000 person-years (PY) [7–11]. Compared with HIV-negative individuals, relative risks of CVD incidence are generally 1.5- to 2-fold greater for PLWH [12]. In a meta-analysis of 793,635 individuals with a total of 3.5 million PY of follow up, the global burden of HIV-associated CVD tripled over the past 2 decades and accounted for 2.6 million disability-adjusted life-years per year [12]. Therefore, investigating the risk factors of CVD among PLWH is essential and imperative as it remains a serious non-AIDS event and contributes significantly to all-cause mortality.

The CVD burden among PLWH as a health issue of growing concern could be attributed to both HIV-related and conventional risk factors. Factors that may contribute to the development of CVD among PLWH include certain HIV treatment regimens (for example, some specific ART drugs/classes including protease inhibitors, integrase strand-transfer inhibitors, and nonnucleoside reverse-transcriptase inhibitors) [13–15], as well as HIV-related clinical responses such as low most recent or nadir CD4 counts and chronic inflammation related to ongoing HIV replication [16, 17]. Non-suppressed HIV viral load due to the lack of retention in care in PLWH is also associated with elevated CVD risk, which suggests that HIV treatment cascade factors play an important role in the development of CVD [18]. In addition, among conventional risk factors, aging is an important factor as an increasing number of CVD events occur among older PLWH [19]. Cigarette smoking and recreational drug use (e.g., cocaine and amphetamine) as two conventional risk factors may augment CVD risk among PLWH [20–22]. Likewise, cardiometabolic risk factors such as hypertension, dyslipidemia, and diabetes are associated with higher risk of CVD events among PLWH [16, 23–25]. Although these conventional risk factors would also increase CVD risks among non PLWH, the prevalence of these risk factors is higher among PLWH [26]. 

While many existing studies investigated the risk factors of CVD incidence among PLWH, several knowledge gaps remain. First, considering the impact of the advances in HIV treatment and the change in age composition of PLWH [19], there was a lack of evidence from relevant studies regarding the dynamics in risk factors associated with the development of CVD. Second, many existing studies had methodological limitations such as data incompleteness and short follow-up time. For example, the total follow-up time was limited to no more than 10 years in two studies [27, 28]. Moreover, there is limited research on how risk factors for CVD and the impact of these factors on CVD incidence among PLWH has changed over time.

To address these knowledge gaps, we conducted a population-based statewide study to examine CVD incidence and risk factors in PLWH by using data retrieved from an integrated electronic health record (EHR) in South Carolina (SC). In the present study, we aimed to: (1) determine the incidence of CVD among PLWH; and (2) investigate the conventional and HIV-related risk factors with CVD incidence and how the impact of these factors on CVD incidence change over time.

## Method

### Study design and population

This study utilized integrated EHR data from a retrospective population-based cohort in SC, which include the enhanced HIV/AIDS reporting system (e-HARS) from SC Department of Health and Environmental Control (DHEC) and Uniform Billing (UB) form format (UB92 or UB04) claim data from SC Revenue and Fiscal Affairs Office (RFA). The e-HARS includes HIV/AIDS case reports with CD4 counts and viral loads records since 2004. The RFA, a state agency playing the role of honest data broker in the study, provides individual-level healthcare visit information. All the chronic condition diagnoses information were collected in the UB claim data. Details of the research protocol have been described elsewhere [29]. 

Our study cohort included the statewide individuals with a confirmed HIV diagnosis between January 01, 2006 and December 31, 2019. We restricted our study population to those at least 18 years old with a valid SC residency status at the time of HIV diagnosis and having at least one CD4 and one viral load (VL) laboratory test in their EHR. PLWH who were diagnosed with CVD before the HIV diagnosis were excluded from the cohort.

To explore the dynamics in risk factors associated with CVD incidence, the analysis was repeated among three groups. The group 1 (*n* = 8,938) included all the eligible individuals (with at least one CD4 and VL test during the follow-up period), yet only the first 5-years of follow up records after HIV diagnosis (follow-up period ranged from 0 to 5 years) was used for analysis. In the second group (group 2, *n* = 4,255), we restricted PLWH to those who did not develop CVD diagnosis in first 5 years and had follow up records (i.e., at least one CD4 count and VL test) during 6–10 years of follow up. Using similar inclusion criteria, in the third group (group 3, *n* = 1,592), we further restricted PLWH to those who had at least 11 years of follow-up record (we used 11–15 years of follow up record for analysis) but did not have CVD diagnosis throughout the first 10 years of HIV diagnosis. The starting year in each group (e.g., 1st, 6th, or 11th ) was considered as “baseline” during the group analysis.

### Outcomes

The primary outcome, CVD event, was defined as the first occurrence of CVD after HIV diagnosis and was measured based on the presence of International Classification of Diseases (ICD)-9 and ICD-10 diagnostic codes. The detailed diagnostic codes are listed in Supplement Table S1. In addition, CVD incidence rate (per 1,000 PY) was defined as the number of CVD events divided by the total person year (PY) at risk. The total PY at risk was calculated by the sum of the individual follow-up period in years. The follow-up period started from the date of HIV diagnosis and ended on the day of death, time of the first occurrence of CVD, or the end of data point in the study (December 31, 2020), whichever occurred first.

### Predictors

#### Demographic characteristics

Demographic characteristics of the cohort included age at HIV diagnosis (18–29, 30–39, 40–49, 50–59, and ≥ 60 years), sex (e.g., male, female), race/ethnicity (e.g., White, Black), and residence type (i.e., urban, rural). All of these variables were treated as binary or categorical variables in the overall and group analyses.

#### Conventional CVD risk factors

The conventional CVD risk factors consisted of substance use, cardiometabolic risk factors, and comorbidities. These conventional CVD risk factors were defined based on the corresponding ICD-9 and ICD-10 diagnosis codes. The substance use included tobacco use and alcohol use. The cardiometabolic risk factors composed of hypertension, diabetes mellitus (DM), and dyslipidemia. The comorbidity risk factors incorporated obesity and chronic kidney disease (CKD). These factors were treated as time-varying variables in all the analyses. In addition, family history of CVD before HIV diagnosis was included as a time-independent risk factor. All these risk factors were treated as binary variables (yes/no).

#### HIV-related factors

The HIV-related factors include HIV transmission mode, immunological indicator, and virological indicator. First, HIV transmission mode included men who have sex with men (MSM) or injection drug use (IDU). Second, CD4 count was used to generate four indicators to monitor the immunologic status among PLWH. These indicators included the “first CD4 counts” at the starting time of the follow-up period, “recent CD4 counts” before the end date of follow up, and the “percentage of days with low CD4 counts (< 500 cells/mm^3^)”. In addition, the “initial CD4 counts” were included to represent the first CD4 counts at HIV diagnosis. For the purpose of data analysis in the current study, all CD4 counts related variables were converted to categorical variables as < 200, 200–350, and > 350 cells/mm^3^. The percentage of days with low CD4 counts was categorized as 0–25%, 26–70% and 71–100%. Third, the percent of days with viral suppression [< 200 copies/ml] was used to indicate the virological status. This variable was also treated as a categorical variable (0–20%, 21–50%, and 51–100%). Finally, the clinical AIDS diagnosis at the time of HIV diagnosis and the year of HIV diagnosis were also included as predictors. Except for HIV transmission mode, AIDS status, and year of HIV diagnosis, all these HIV-specific factors were treated as time-varying variables in the analysis.

#### HIV treatment cascade factors

Two predictors related to HIV care engagement, timely linkage to care and rate of retention in care, were included in the analysis. Timely linkage to care was defined as the having first CD4 cell count test or viral load test in no more than 30 days after HIV diagnosis. Percent of years in retention in care was defined as the years in care divided by the total follow-up years. Retention in care was defined as having either at least two CD4 cell counts or two viral load measurements, separated by at least 90 days within a follow-up year. Percent of years in retention in care was treated as time-varying variable and categorized into three levels: 0–30%, 31-75%, 76-100%.

### Statistical analysis

We applied mean and standard deviation (SD) for continuous variables, and number and percentage for categorical variables. Chi-square test was used to test the difference by incidence of CVD in demographic and other predictors. T-test was used to test the difference in HIV diagnosis year. Multivariable logistic regression was used to assess the relationship between incidence of CVD and all risk factors for overall population and three groups with different follow-up periods. *P*-value less than 0.05 were considered significant. The forest plots with adjusted Odds Ratio (aOR) and 95% confidence interval (CI) were displayed to present the association between risk factors and CVD incidence during different follow up periods. All analyses were performed using SAS version 9.4 (SAS Institute Inc., Cary, North Carolina, USA) and R software version 4.4.2.

**Fig. 1:**
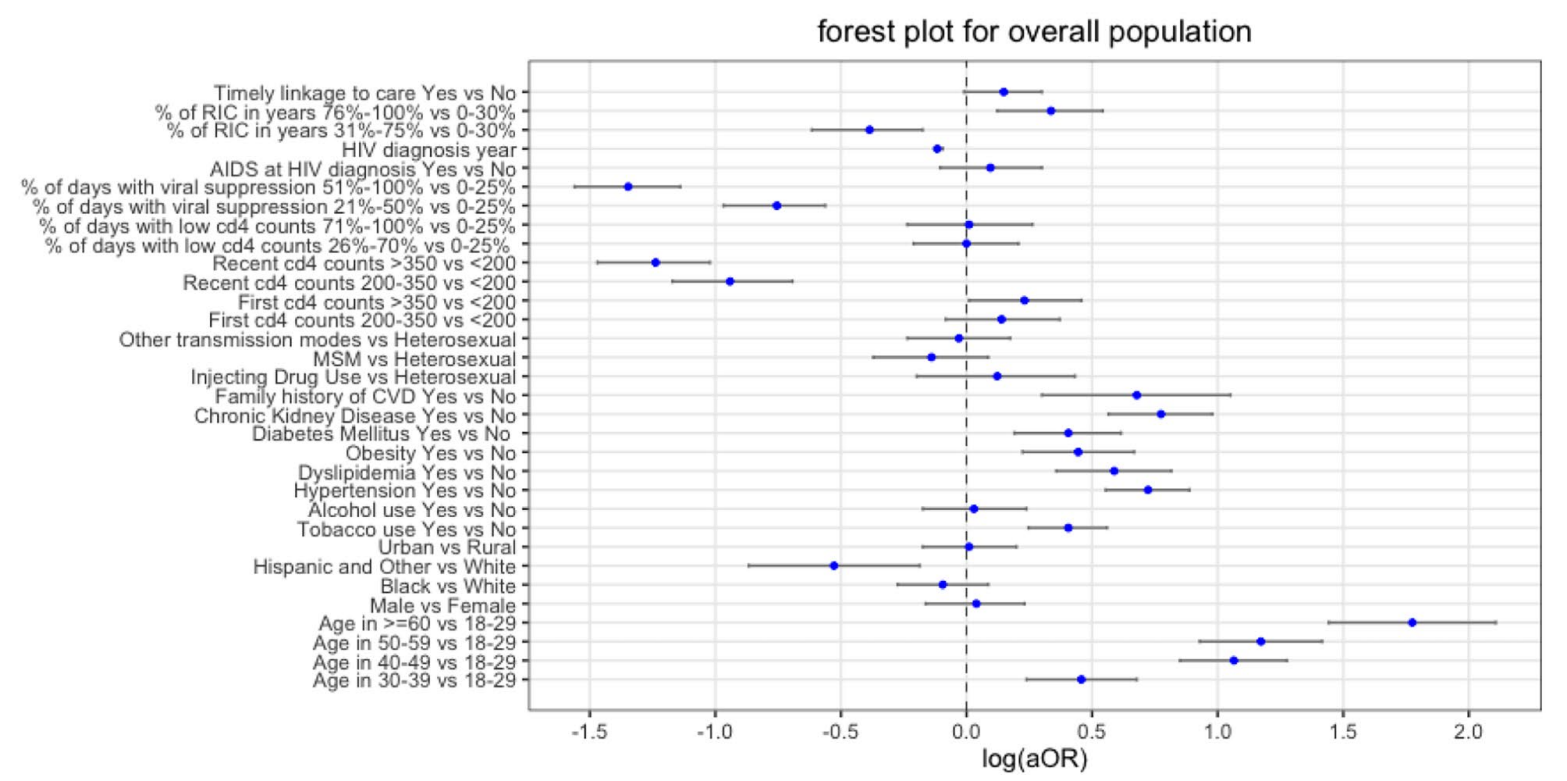
Forest plot about logarithmic aOR value of each risk factors included in the logistic regression for overall population Note: “RIC” refers to retention in care

## Results

### Characteristics of study population

The study population consisted of 9,082 PLWH with a mean age of 34.7 years old at HIV diagnosis. Over half of the study population were in age groups 18–29 and 30–39 years (44.14% & 21.88%). The majority of the study population was male (77.32%), Black (69.04%), or from rural areas (83.36%). The CVD prevalence was 12.92%. As shown in Table 1, a total of 1,173 PLWH experienced CVD over time. The incidence rate of CVD was 18.64 cases per 1000 PY among a total of 62,924.2 PY follow-up.

**Table 1:** Baseline characteristics among overall population and patients grouped by CVD

Characteristic	Overall	CVD	
	*N* = 9,082	No 7,909(87.08%)	Yes 1,173(12.92%)
Age at HIV diagnosis (years)			
18–29	4,009 (44.14)	3,778 (47.77)	231 (19.69)
30–39	1,987 (21.88)	1,772 (22.4)	215 (18.33)
40–49	1,759 (19.37)	1,388 (17.55)	371 (31.63)
50–59	1,017 (11.2)	767 (9.7)	250 (21.31)
>=60	310 (3.41)	204 (2.58)	106 (9.04)
**Sex**			
Female	2,060 (22.68)	1,694 (21.42)	366 (31.2)
Male	7,022 (77.32)	6,215 (78.58)	807 (68.8)
**Race**			
White	2,004 (22.07)	1,756 (22.2)	248 (21.14)
Black	6,270 (69.04)	5,402 (68.3)	868 (74)
Hispanic and Other	808 (8.9)	751 (9.5)	57 (4.86)
**Residence**			
Urban	1,511 (16.64)	1,280 (16.18)	231 (19.69)
Rural	7,571 (83.36)	6,629 (83.82)	942 (80.31)
**Tobacco use**			
No	5,139 (56.58)	4,674 (59.1)	465 (39.64)
Yes	3,943 (43.42)	3,235 (40.9)	708 (60.36)
**Alcohol use**			
No	8,195 (90.23)	7,244 (91.59)	951 (81.07)
Yes	887 (9.77)	665 (8.41)	222 (18.93)
**Hypertension**			
No	6,741 (74.22)	6,202 (78.42)	539 (45.95)
Yes	2,341 (25.78)	1,707 (21.58)	634 (54.05)
**Dyslipidemia**			
No	8,484 (93.42)	7,514 (95.01)	970 (82.69)
Yes	598 (6.58)	395 (4.99)	203 (17.31)
**Obesity**			
No	8,338 (91.81)	7,349 (92.92)	989 (84.31)
Yes	744 (8.19)	560 (7.08)	184 (15.69)
**Diabetes mellitus**			
No	8,356 (92.01)	7,419 (93.8)	937 (79.88)
Yes	726 (7.99)	490 (6.2)	236 (20.12)
**Chronic kidney disease**			
No	8,435 (92.88)	7,502 (94.85)	933 (79.54)
Yes	647 (7.12)	407 (5.15)	240 (20.46)
**Family history of CVD**			
No	8,823 (97.15)	7,697 (97.32)	1,126 (95.99)
Yes	259 (2.85)	212 (2.68)	47 (4.01)
**HIV transmission risk**			
Heterosexual	1,632 (17.97)	1,311 (16.58)	321 (27.37)
MSM	4,868 (53.6)	4,467 (56.48)	401 (34.19)
Injecting Drug Use	473 (5.21)	380 (4.8)	93 (7.93)
Other	2,109 (23.22)	1,751 (22.14)	358 (30.52)
**First cd4 counts (cells/mm**^**3**^)			
< 200	2,606 (28.69)	2,098 (26.53)	508 (43.31)
200–350	1,917 (21.11)	1,705 (21.56)	212 (18.07)
> 350	4,559 (50.2)	4,106 (51.92)	453 (38.62)
**Recent cd4 counts (cells/mm**^**3**^)			
< 200	1,207 (13.29)	776 (9.81)	431 (36.74)
200–350	1,102 (12.13)	932 (11.78)	170 (14.49)
> 350	6,773 (74.58)	6,201 (78.4)	572 (48.76)
**% of days with low cd4 counts**			
0–25%	3,767 (41.48)	3,485 (44.06)	282 (24.04)
26-70%	2,964 (32.64)	2,537 (32.08)	427 (36.4)
71-100%	2,351 (25.89)	1,887 (23.86)	464 (39.56)
**% of days with viral suppression**			
0–20%	2,915 (32.1)	2,243 (28.36)	672 (57.29)
21-50%	2,168 (23.87)	1,952 (24.68)	216 (18.41)
51-100%	3,999 (44.03)	3,714 (46.96)	285 (24.3)
**AIDS at HIV diagnosis**			
No	7,172 (78.97)	6,375 (80.6)	797 (67.95)
Yes	1,910 (21.03)	1,534 (19.4)	376 (32.05)
**% of years in retention in care**			
0–30%	1,993 (21.94)	1,641 (20.75)	352 (30.01)
31-75%	2,656 (29.24)	2,428 (30.7)	228 (19.44)
76-100%	4,433 (48.81)	3,840 (48.55)	593 (50.55)
**Timely linkage to care**			
No	3,443 (37.91)	3,018 (38.16)	425 (36.23)
Yes	5,639 (62.09)	4,891 (61.84)	748 (63.77)

### Overall risk factors of CVD among PLWH

Risk factors of CVD are reported in Table 2; Fig. 1. Compared with 18–29 years, PLWH with an older age at diagnosis of HIV were more likely to develop CVD (aOR range: 1.58–5.9). Compared to White, Hispanic and other races/ethnicity had a lower risk of CVD (aOR = 0.59, 95%CI: 0.42–0.83). Most of the traditional risk factors, including hypertension (aOR = 2.06, 95% CI: 1.74–2.43), dyslipidemia (aOR = 1.8, 95%CI: 1.43–2.26), CKD (aOR = 2.17, 95% CI: 1.76–2.66), and family history of CVD (aOR = 1.97, 95% CI: 1.35–2.86), were associated with the increased risk of CVD. CVD incidence was lower in those with a higher percentage of days with viral suppression (51-100% vs. 0–20%: aOR = 0.26 95% CI: 0.21–0.32) and higher recent CD4 counts (> 350 cells/mm^3^ vs. < 200 cells/mm^3^: aOR = 0.29 95% CI: 0.23–0.36). In addition, percent of years in retention in care in higher level associated with a lower risk of CVD (31-75% vs. 0-30%: aOR = 0.68, 95% CI: 0.54–0.84). It is worth noting that individuals with an initial CD4 count > 350 cells/mm^3^ has increased risk of CVD compared with those whose initial CD4 count was < 200 cells/mm^3^ (aOR = 1.26, 95%: 1.01–1.58).

**Table 2:** Multivariable logistic regression analysis for variables among overall population

Effect	aOR	*p*-value
Age in 30–39 vs. 18–29	1.58 (1.27, 1.97)	< 0.0001
Age in 40–49 vs. 18–29	2.9 (2.34, 3.58)	< 0.0001
Age in 50–59 vs. 18–29	3.23 (2.53, 4.12)	< 0.0001
Age in > = 60 vs. 18–29	5.9 (4.23, 8.23)	< 0.0001
Male vs. Female	1.04 (0.85, 1.26)	0.721
Black vs. White	0.91 (0.76, 1.09)	0.3149
Hispanic and Other vs. White	0.59 (0.42, 0.83)	0.0023
Urban vs. Rural	1.01 (0.84, 1.22)	0.9185
Tobacco use Yes vs. No	1.5 (1.28, 1.75)	< 0.0001
Alcohol use Yes vs. No	1.03 (0.84, 1.27)	0.7556
Hypertension Yes vs. No	2.06 (1.74, 2.43)	< 0.0001
Dyslipidemia Yes vs. No	1.8 (1.43, 2.26)	< 0.0001
Obesity Yes vs. No	1.56 (1.25, 1.95)	< 0.0001
Diabetes Mellitus Yes vs. No	1.5 (1.21, 1.85)	0.0002
Chronic Kidney Disease Yes vs. No	2.17 (1.76, 2.66)	< 0.0001
Family history of CVD Yes vs. No	1.97 (1.35, 2.86)	0.0004
Injecting Drug Use vs. Heterosexual	1.13 (0.82, 1.54)	0.4626
MSM vs. Heterosexual	0.87 (0.69, 1.09)	0.2196
Other transmission modes vs. Heterosexual	0.97 (0.79, 1.19)	0.794
First cd4 counts (cells/mm^3^) 200–350 vs. < 200	1.15 (0.92, 1.45)	0.2219
First cd4 counts (cells/mm^3^) > 350 vs. < 200	1.26 (1.01, 1.58)	0.0432
Recent cd4 counts (cells/mm^3^) 200–350 vs. < 200	0.39 (0.31, 0.5)	< 0.0001
Recent cd4 counts (cells/mm^3^) > 350 vs. < 200	0.29 (0.23, 0.36)	< 0.0001
% of days with low cd4 counts 26-70% vs. 0–25%	1 (0.81, 1.23)	0.9962
% of days with low cd4 counts 71-100% vs. 0–25%	1.01 (0.79, 1.3)	0.9136
% of days with viral suppression 21-50% vs. 0–20%	0.47 (0.38, 0.57)	< 0.0001
% of days with viral suppression 51-100% vs. 0–20%	0.26 (0.21, 0.32)	< 0.0001
AIDS at HIV diagnosis Yes vs. No	1.1 (0.9, 1.35)	0.3586
HIV diagnosis year	0.89 (0.88, 0.91)	< 0.0001
% of years in retention in care 31-75% vs. 0–30%	0.68 (0.54, 0.84)	0.0006
% of years in retention in care 76-100% vs. 0–30%	1.4 (1.13, 1.72)	0.0018
Timely linkage to care Yes vs. No	1.16 (0.99, 1.35)	0.0636

### Dynamics in risk factors

To explore the dynamics in risk factors associated with incidence of CVD, three multivariable logistic regression models are respectively applied to the Groups 1–3 (0–5 yrs, 6–10 yrs and 11–15 yrs of follow-up) (Table 3, Supplement Fig. 1). Traditional risk factors in the 5-year and 10-year follow-up groups remained the same significance as the overall group. However, the effect of some factors such as dyslipidemia, obesity and CKD faded away in the 10-year group compared to that in the 5-year group. Tobacco use, hypertension, obesity, and CKD kept associated with CVD in the 15-year group.

**Table 3:** Multivariable logistic regression analysis for variables among three groups

Effect	Group1(*N*=8938)		Group2(*N*=4255)		Group3(*N*=1592)	
	aOR	*p*-value	aOR	*p*-value	aOR	*p*-value
Age in 30-39 vs. 18-29	1.45 (1.1,1.92)	0.0084	2.02 (1.32,3.07)	0.0011	1.72 (0.85,3.48)	0.1338
Age in 40-49 vs. 18-29	2.48 (1.9,3.22)	<0.0001	3.16 (2.09,4.77)	<0.0001	3.22 (1.63,6.38)	0.0008
Age in 50-59 vs. 18-29	2.92 (2.19,3.91)	<0.0001	3.62 (2.2,5.96)	<0.0001	4.71(2.07,10.71)	0.0002
Age in >=60 vs. 18-29	5.05 (3.47,7.35)	<0.0001	8.68 (4.14,18.2)	<0.0001	3.56(0.82,15.41)	0.09
Male vs. Female	0.98 (0.78,1.24)	0.88	1.25 (0.85,1.83)	0.2601	1.21 (0.67,2.17)	0.5315
Black vs. White	0.89 (0.71,1.12)	0.3229	1.08 (0.75,1.56)	0.6862	0.71 (0.4,1.28)	0.2523
Hispanic and Other vs. White	0.7 (0.48,1.03)	0.0724	0.54 (0.22,1.37)	0.1978	0.71 (0.21,2.37)	0.5753
Urban vs. Rural	1.11 (0.89,1.39)	0.3451	1.22 (0.87,1.71)	0.2447	0.56 (0.29,1.08)	0.0851
Tobacco use Yes vs. No	1.64 (1.36,1.97)	<0.0001	1.68 (1.24,2.28)	0.0008	1.87 (1.11,3.16)	0.0185
Alcohol use Yes vs. No	1.16 (0.89,1.5)	0.2651	0.91 (0.62,1.33)	0.6224	1.17 (0.67,2.04)	0.5738
Hypertension Yes vs. No	2.1 (1.71,2.57)	<0.0001	2.12 (1.54,2.91)	<0.0001	2.52 (1.49,4.24)	0.0005
Dyslipidemia Yes vs. No	2.52 (1.85,3.42)	<0.0001	1.85 (1.25,2.74)	0.0022	1.26 (0.69,2.31)	0.4532
Obesity Yes vs. No	1.83 (1.36,2.46)	<0.0001	1.59 (1.07,2.37)	0.0226	2.01 (1.14,3.55)	0.0163
Diabetes Mellitus Yes vs. No	1.52 (1.16,1.99)	0.0021	2.09 (1.45,3)	<0.0001	1.21 (0.67,2.2)	0.5262
Chronic Kidney Disease Yes vs. No	3.13 (2.41,4.07)	<0.0001	2.19 (1.51,3.17)	<0.0001	2.36 (1.29,4.28)	0.005
Family history of CVD Yes vs. No	1.71 (1.12,2.62)	0.0134	2.85 (1.34,6.09)	0.0067	——	——
Injecting Drug Use vs. Heterosexual	1.38 (0.95,2)	0.0939	0.95 (0.51,1.76)	0.8648	0.7 (0.24,2.01)	0.5061
MSM vs. Heterosexual	0.94 (0.7,1.24)	0.6446	1.09 (0.71,1.68)	0.6869	0.61 (0.3,1.24)	0.173
Other transmission modes vs. Heterosexual	0.94 (0.73,1.2)	0.6191	0.94 (0.63,1.39)	0.7488	1.16 (0.65,2.07)	0.6111
First cd4 counts (cells/mm^3^) 200-350 vs. <200	1.23 (0.92,1.63)	0.1643	0.88 (0.53,1.44)	0.6058	1.16 (0.48,2.83)	0.7379
First cd4 counts (cells/mm^3^) >350 vs. <200	1.28 (0.95,1.72)	0.1054	0.83 (0.49,1.42)	0.4998	0.64 (0.23,1.79)	0.3972
Initial cd4 counts (cells/mm^3^) 200-350 vs. <200	——	——	1.19 (0.79,1.79)	0.4085	0.62 (0.3,1.25)	0.1788
Initial cd4 counts (cells/mm^3^) >350 vs. <200	——	——	1.15 (0.77,1.7)	0.4996	0.81 (0.44,1.49)	0.5003
Recent cd4 counts (cells/mm^3^) 200-350 vs. <200	0.36 (0.27,0.47)	<0.0001	0.34 (0.21,0.56)	<0.0001	0.34 (0.14,0.84)	0.0195
Recent cd4 counts (cells/mm^3^) >350 vs. <200	0.22 (0.17,0.3)	<0.0001	0.18 (0.11,0.29)	<0.0001	0.25 (0.1,0.6)	0.0019
% of days with low cd4 counts 26-70% vs. 0-25%	1.02 (0.78,1.35)	0.8673	0.81 (0.53,1.24)	0.3392	0.69 (0.34,1.41)	0.3056
% of days with low cd4 counts 71-100% vs. 0-25%	0.92 (0.67,1.27)	0.6208	0.17 (0.08,0.38)	<0.0001	——	——
% of days with viral suppression 21-50% vs. 0-20%	0.44 (0.34,0.57)	<0.0001	0.5 (0.33,0.74)	0.0007	1.42 (0.73,2.77)	0.3044
% of days with viral suppression 51-100% vs. 0-20%	0.27 (0.21,0.34)	<0.0001	0.51 (0.35,0.75)	0.0006	1.16 (0.65,2.08)	0.6239
AIDS at HIV diagnosis Yes vs. No	1.33 (1.04,1.69)	0.0234	0.93 (0.63,1.38)	0.7135	0.44 (0.21,0.92)	0.0288
HIV diagnosis year	1 (0.97,1.02)	0.6538	0.8 (0.76,0.85)	<0.0001	0.55 (0.45,0.67)	<0.0001
% of years in retention in care 31-75% vs. 0-30%	0.52 (0.39,0.69)	<0.0001	0.57 (0.36,0.9)	0.017	0.24 (0.09,0.6)	0.0026
% of years in retention in care 76-100% vs. 0-30%	0.86 (0.68,1.1)	0.2371	0.93 (0.64,1.36)	0.7215	0.85 (0.43,1.66)	0.6287
Timely linkage to care Yes vs. No	1.33 (1.09,1.61)	0.0047	1 (0.75,1.33)	0.9866	1.41 (0.87,2.29)	0.1601

Note: For model corresponding to group1, only the variable first CD4 counts was remained due to the equality between initial CD4 counts and first CD4 counts. In addition, because there are no individuals belonging to the level of 71-100% for variable percent of low CD4 counts in group 3, the level is not included in the corresponding model

For the HIV specific factors, recent CD4 counts 200–350 cells/mm^3^ and > 350 cells/mm^3^ remained significantly negative effect against CVD during all period of follow-up with aOR range 0.34–0.36 and 0.18–0.25, respectively. The effect of percent of days with viral suppression 21-50% and 51-100% was significant only for 5-year follow-up group and 10-year follow-up group with aOR range 0.44–0.5 and 0.27–0.51, respectively. Among HIV treatment cascade factors, percent of years in retention in care in level 31–75% remained significant during each group analysis (aOR range: 0.24–0.57). Notably, timely linkage to care presented an increased risk of CVD in group 1 (aOR = 1.33, 95% CI: 1.09–1.61).

## Discussion

Using the integrated EHR data in SC, this study examined the incidence rate of CVD and conventional and HIV-related risk factors of CVD. More importantly, our study highlighted the changes in association between risk factors and CVD incidence during different follow-up periods. While most conventional and HIV-related factors were only significantly associated with the incidence of CVD during the 5-year and 10-year follow-ups, factors including hypertension, obesity, CKD, recent CD4, and percent of years in retention in care had a long-term impact on CVD incidence. With a larger sample size and longer follow up record, the utilization of statewide EHR from multiple sources enabled us to examine the dynamic risk factors related to the occurrence of CVD among PLWH over time.

The incidence rate of CVD (18.64 cases per 1,000 person-year) in our study was higher when compared to the findings from other studies (2.57–4.70 cases per 1,000 person-year) [7, 9–11]. The discrepancy may be attributed to the following reasons. First, the 15 years follow up period in our study, is longer than some of other studies which have follow-up durations of no more than 10 years [11]. The CVD incidence rate would increase as the follow-up time extends [5]. Second, the difference in the definition of CVD could lead to inconsistency as our study identified and included a broad spectrum of CVD events [5, 7, 10]. 

The wide application of ART had increased life expectancy among PLWH [30]. However, the increased life expectancy was also accompanied by elevated risk of CVD caused by aging process. Compared with CVD-free PLWH, individuals with CVD had a greater proportion of chronic diseases including DM, CKD, hypertension, and obesity. Moreover, the findings from the overall population suggested a significant association between these chronic diseases and the incidence of CVD. These findings were consistent with the general population [16, 24, 31]. 

Higher recent CD4 counts and percent of days with viral suppression were two strong HIV-related factors that had a protective effect on CVD among PLWH. This was consistent with past studies [32–34]. These findings have highlighted the importance of maintaining a high CD4 count and optimal viral suppression to prevent the CVD occurrence.

By analyzing CVD incidence over three groups, we were able to demonstrate that the impact of various risk factors varies over time. Among those conventional risk factors, tobacco use, hypertension, obesity, and CKD had a persistent association with CVD. It was worth noting that alcohol use did not show significance during any follow-up period, which differs from previous studies findings [35]. For HIV-related factors, higher recent CD4 counts revealed a protective effect on the incidence of CVD during all follow-up periods. The finding corroborated with findings in other studies which found that low CD4 count was a significant risk factor for CVD among PLWH [16, 33, 34, 36]. In addition, long-lasting duration of viral suppression (i.e. percentage of days with viral suppression) was indicated to reduce the risk of CVD only in the middle and short term (only significant for group 1 and 2) but such effect might diminish in the long term.

In terms of HIV care engagement variables, the percent of years in retention in care in mid-level (31-75% vs. 0–30%) had a long-term preventive impact on the incidence of CVD while timely linkage to care only had a short-term effect on the incidence of CVD (only significant for group 1). The protective effect between percent of years in retention in care and incidence of CVD could be attributed to the reason that most individuals who often participate in the HIV care tend to have a more active self-health management for relevant comorbidities including CVD [37, 38]. 

There are several limitations in the current study. First, both the outcome variable, the first onset of CVD events, and several important predictor variables were only defined based on the ICD-9/10 codes without using other clinical information (e.g., lab, procedures, medications, medical notes) due to data accessibility issues. Solely reliance on ICD codes to define CVD lacks corroboration from other validation datasets such as the clinical data and medical diagnostic records towards each patient, which would result in the loss of validity and accuracy in relevant conclusions [39]. Nevertheless, previous literatures demonstrated acceptable or higher sensitivity in solely reliant on ICD codes to define similar variables [40, 41]. Therefore, we think the potential misclassification bias of both outcome measure and certain predictors is not substantial. Second, some important risk factors related to CVD among PLWH were either unavailable or missing in the dataset, such as physical activities and ART information. The ART (e.g., integrase strand-transfer inhibitors) toxicity might be related to the CVD development [15]. Third, with the extension of follow-up time, the number of patients in group 3 significantly decreased. Such a decrease could result in the loss of precision on model estimations, as observed from the wider confidence interval in the forest plots (Supplement Fig. 1). Fourth, since the assumption of Cox proportional hazard model does not satisfy, we applied logistic regression instead of Cox regression to model time to CVD. Although odds ratios (ORs) derived from logistic regression may overestimate the risk of incident CVD, considering only first CVD occurrence was considered and no recurrence is taken into account, we believed ORs is more appropriate than the relative risks derived from robust Poisson regression for the interpretation purpose [42]. Finally, because the data sources were from SC, we should be cautious about generalizing the findings to other states.

## Conclusions

In conclusion, our study investigated the predictors of CVD incidence among PLWH and compared the temporal change of these risk factors. More personalized, dynamic prevention and treatment of CVD as well as HIV care management are needed especially considering the changing impact of different factors over time. More specifically, the exposure to most conventional CVD risk factors including smoking and obesity should be monitored, prevented, and managed throughout the entire life course of HIV infection. For prevention of CVD among PLWH who have been diagnosed for a long time, we should focus on restoring immune recovery and improving retention in care. The findings from our study could inform future research to focus on the change in the association between risk factors and incidence of CVD over time, use different data sources to verify our findings, and supplement more information on dynamic analysis of CVD risk factors.

### Electronic supplementary material

Below is the link to the electronic supplementary material.

Supplementary Material 1

## Data Availability

The University of South Carolina is prohibited from making individual-level data available publicly due to provisions in our data use agreements with state agencies/data providers, institutional policy, and ethical requirements. To facilitate research, we make access to such data available via approved data access requests. The data is unavailable externally or for re-release due to prohibitions in data use agreements with the South Carolina Department of Health and Environmental Control (SC DHEC). For more information or guidance on how to make a request, please contact (Bankole Olatosi, PhD): Olatosi@mailbox.sc.edu. The underlying analytical codes are available from the authors on request.

## Abbreviations

CVD: cardiovascular disease
PLWH: people living with HIV
CKD: chronic kidney disease
ART: antiretroviral therapy
PY: person-years
EHR: electronic health record
SC: South Carolina
e-HARS: enhanced HIV/AIDS reporting system
DHEC: department of health and environmental control
UB: Uniform Billing
RFA: revenue and fiscal affairs office
VL: viral load
DM: diabetes mellitus
ICD: international classification of diseases
MSM: men who have sex with men
IDU: injection drug use
SD: standard deviation
aOR: adjusted odds ratio
CI: confidence interval
