# Supplementary material for: The dynamic risk factors of cardiovascular disease among people living with HIV: a real-world data study
Source: BMC Public Health. 2024 Apr 25;24:1162. doi: 10.1186/s12889-024-18672-x (PMC11044498; doi:10.1186/s12889-024-18672-x)
Supplement: Supplementary file 1 — Supplementary Material 1 [file 12889_2024_18672_MOESM1_ESM.docx]

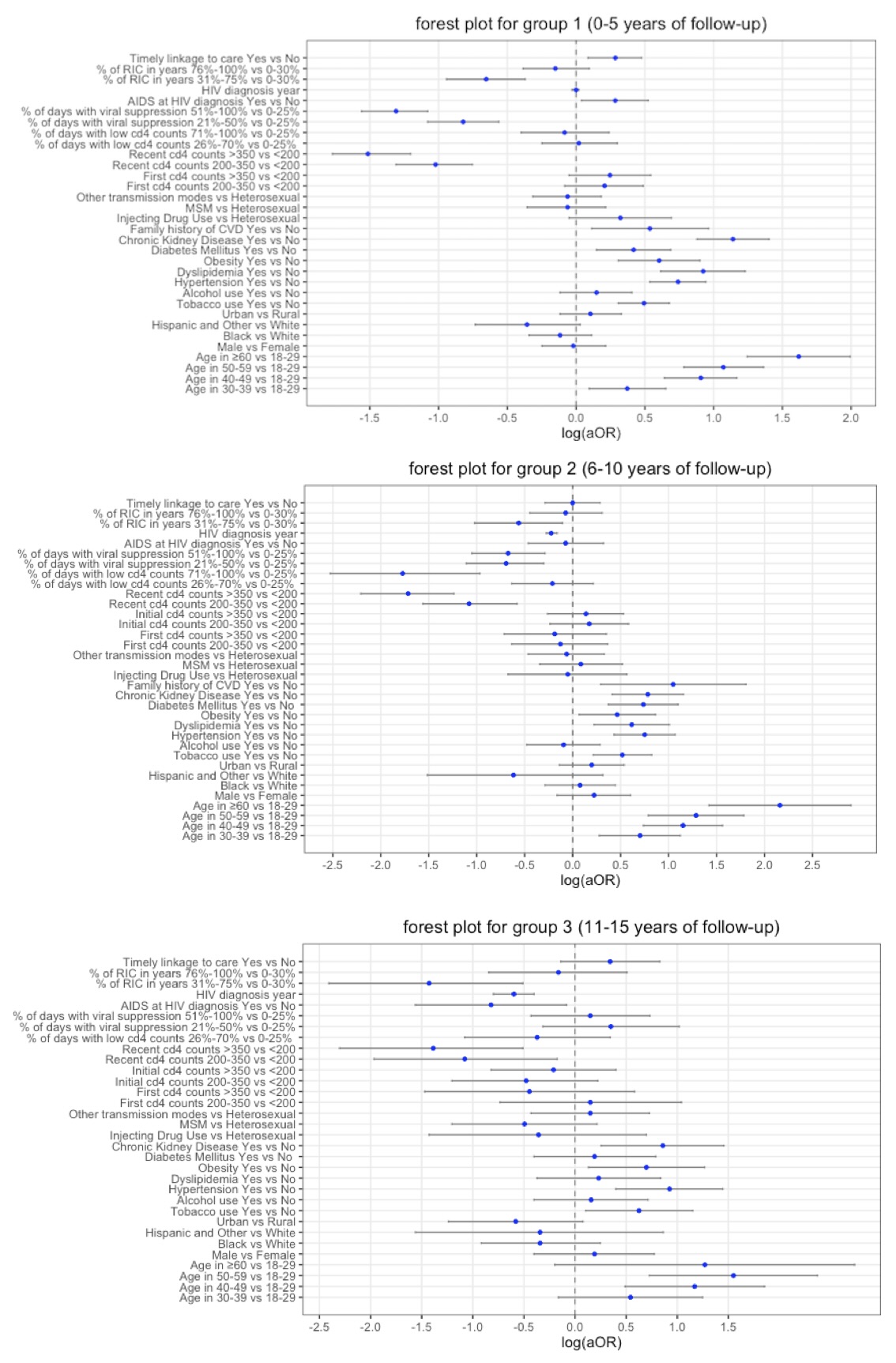


Note: “RIC” refers to retention in care

Supplement Figure 1. forest plot about logarithmic aOR value of each risk factors included in the logistic regression for individuals in group 1, group 2, and group 3

Supplement Table S1. CVD measures based on ICD-9/10 codes

| Outcomes | Diagnosis | ICD-9 | ICD-10 |
| --- | --- | --- | --- |
| CVD | Coronary heart disease (CHD) | 410–414, 427.5, 429.2, 798.1, 798.2 and 798.9 | I20–I26, I46 and R96 |
|  | Cerebrovascular disease | 342, 430–438 | I60–I67, I69, G45 |
|  | Heart failure | 428 | I11.0, I13.0, I13.2, I50 |
|  | Atherosclerosis, aortic aneurysm and dissection, other aneurysm, other peripheral vascular diseases and arterial embolism and thrombosis | 440–442, 443.9, 444 | I70–I74 |
|  | Other cardiovascular disease | 402, 416, 424, 425, 427, 429 | I11.9, I27, I35, I38, I39, I42, I43, I47, I48.9, I49, I51 |
